# A Case of Traumatic Diaphragm Rupture: A Differential Diagnosis Not to Be Missed

**DOI:** 10.7759/cureus.49820

**Published:** 2023-12-02

**Authors:** Dunya Alfaraj, Khaleel I Alwatyan, Mustafa H Al Ashour, Faisal A Alghamdi, Abdullah A Alhowaish

**Affiliations:** 1 Emergency Medicine, Imam Abdulrahman Bin Faisal University, King Fahad University Hospital, Dammam, SAU; 2 Medicine, Imam Abdulrahman Bin Faisal University, King Fahad University Hospital, Dammam, SAU

**Keywords:** sob - shortness of breath, emergency medicine and trauma, fall from height, major trauma, traumatic diaphragmatic hernia

## Abstract

Traumatic diaphragmatic rupture is a rare condition with clinical stigmata that overlaps with a similarly fatal condition such as tension pneumothorax. Although the former is much rarer, early differentiation between a ruptured diaphragm and a tension pneumothorax is crucial to avoid incorrect interventions. In this case, we present a middle-aged male construction worker who fell from the roof of a two-story building and presented to our emergency department with a clinical presentation similar to that of tension pneumothorax. However, a chest X-ray later revealed a left diaphragmatic hernia, which completely altered the management. This case helps highlight the importance of widening one’s list of differential diagnoses, especially in the setting of a hectic environment and a vague presentation.

## Introduction

Diaphragmatic rupture is a rare medical condition that poses challenges in its diagnosis. It occurs in less than 0.5% of all trauma cases, more frequently on the left side (80%) [1]. It is typically caused by various factors, including motor vehicle collisions, falls from heights, stabbings, gunshot wounds, missile injuries, or incidents during chest or abdominal surgeries. Among these causes, motor vehicle collisions are the most prevalent [1-3].

The pathophysiology of diaphragmatic hernia includes circulatory and respiratory depression secondary to decreased diaphragmatic function. Herniation of intra-abdominal contents into the thorax leads to cardiac and pulmonary compression, causing a mediastinal shift to the opposite side [4]. The stomach is the most commonly herniated organ, followed by the colon, and then the spleen [5].

The pathophysiological consequences create symptoms that may overlap with pneumothorax, leading to diagnostic challenges and necessitating clinicians to maintain a high level of suspicion for a ruptured diaphragm. These shared symptoms include chest pain, cough, tachycardia, dyspnea, cyanosis, and the absence of breath sounds over the affected hemithorax [6-9]. There are no specific signs of a diaphragmatic rupture. However, certain signs lean more toward it, such as bowel sounds heard over the affected hemithorax, palpable abdominal contents upon insertion of a chest tube, paradoxical movements of the abdomen with breathing, and a less full abdomen on palpation [10].

Early differentiation between a ruptured diaphragm and pneumothorax is crucial to avoid incorrect interventions, such as inserting a chest tube that could potentially damage a herniated organ [8,9]. Since a significant force is required to rupture the diaphragm, it is uncommon for this to occur in isolation. Instead, it is often found in conjunction with other injuries, such as head, chest, and abdominal injuries, which are the primary contributors to mortality, rather than the diaphragmatic rupture alone [11]. Consequently, this condition indicates severe trauma, and when diagnosed, medical professionals should be prepared to examine and address potential accompanying injuries.

The diagnosis of a ruptured diaphragm heavily relies on a radiographic examination. Keeping this diagnosis in mind, a chest X-ray is necessary when there is suspicion of traumatic diaphragmatic hernia; however, it cannot be relied on for diagnosis. Diagnostic tools used to diagnose a ruptured diaphragm include chest radiographs, CT scans, ultrasound, diagnostic laparoscopy, and/or video-assisted thoracoscopic surgery (VATS) [2].

In this case report, we present a rare emergency scenario: traumatic diaphragmatic rupture with transthoracic abdominal organ herniation, along with a literature review. The aim is to raise awareness among emergency physicians about the significance of considering a ruptured diaphragm as a potential diagnosis alongside pneumothorax, even though it is rare. By highlighting this condition, we hope to emphasize the importance and pathway of early and accurate differentiation between the two, ensuring appropriate and timely interventions for better patient outcomes.

## Case presentation

A 48-year-old male construction worker with no medical history was brought to the emergency department by ambulance after sustaining a fall from the roof of a two-story building. Upon arrival, the patient's vitals were taken. His pulse was at 139 beats per minute, the respiratory rate was 22 breaths per minute, the blood pressure was 95/67 mmHg, the oxygen saturation was 88% on room air, and the Glasgow Coma Scale (GCS) was 8/15.

**Figure 1:**
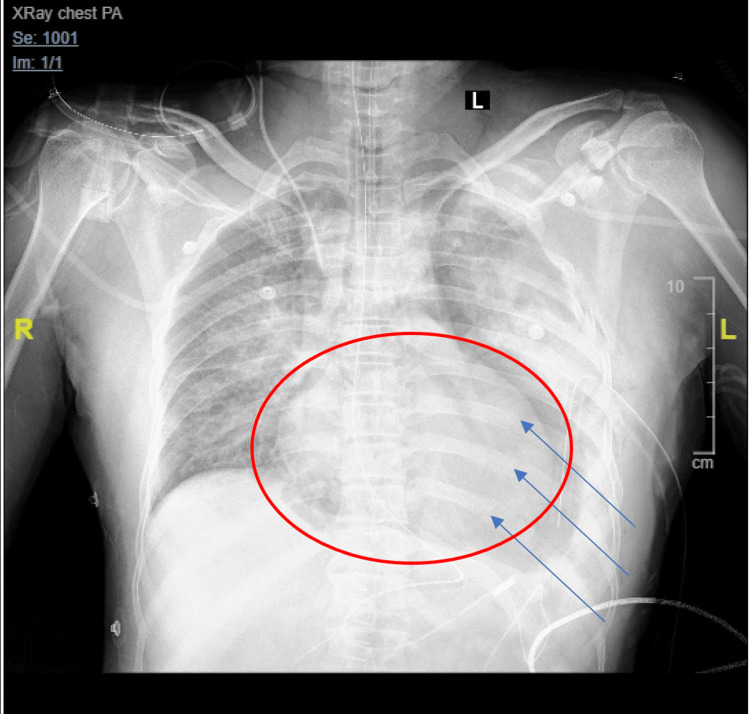
An anterior-posterior chest radiograph shows a left diaphragmatic hernia with protrusion of internal abdominal organs into the chest, visualized by the pericardial lucency (red circle) that represents the herniated bowel and multiple left-sided rib fractures (blue arrows) with underlying hemothorax.

The trauma team was called, and a hard C-collar was immediately applied to the patient, and a bolus of 0.9% of normal saline was given along with two units of uncrossed O+-packed red blood cells. During the primary survey, the patient began to desaturate to 70% oxygen on room air and was, therefore, quickly intubated. There were no palpable or visual chest movements bilaterally, along with decreased air entry to the left. A chest radiograph was quickly taken, which showed a left diaphragmatic hernia, a left hemothorax, and multiple rib fractures (Figure 1).

The remainder of the primary survey was significant for a lower open fracture of the left tibia.

The relevant laboratory investigations done are tabulated in Table 1.

**Table 1:** Laboratory investigations of the patient pH: acid-base balance; pCO_2_: partial pressure of carbon dioxide; pO_2_: partial pressure of oxygen

Investigation	Result	Reference Range
Arterial Blood Gas	pH: 7.226	7.35 - 7.45
	pCO_2_: 55.3 mmHg	35 - 45 mmHg
	pO_2_: 60.8 mmHg	75 - 100 mmHg
Complete Blood Count	Hemoglobin: 11.5 g/dL	13 - 18 g/dL
	Hematocrit: 35.2%	42-52%
Liver Function Test	Alanine Aminotransferase: 183 U/L	5-34 U/L
	Aspartate Aminotransferase: 102 U/L	5-55 U/L
	Lactate Dehydrogenase: 747 U/L	125-220 U/L
Renal Function Test	Normal	
Amylase	Normal	
Lipase	Normal	

**Figure 2:**
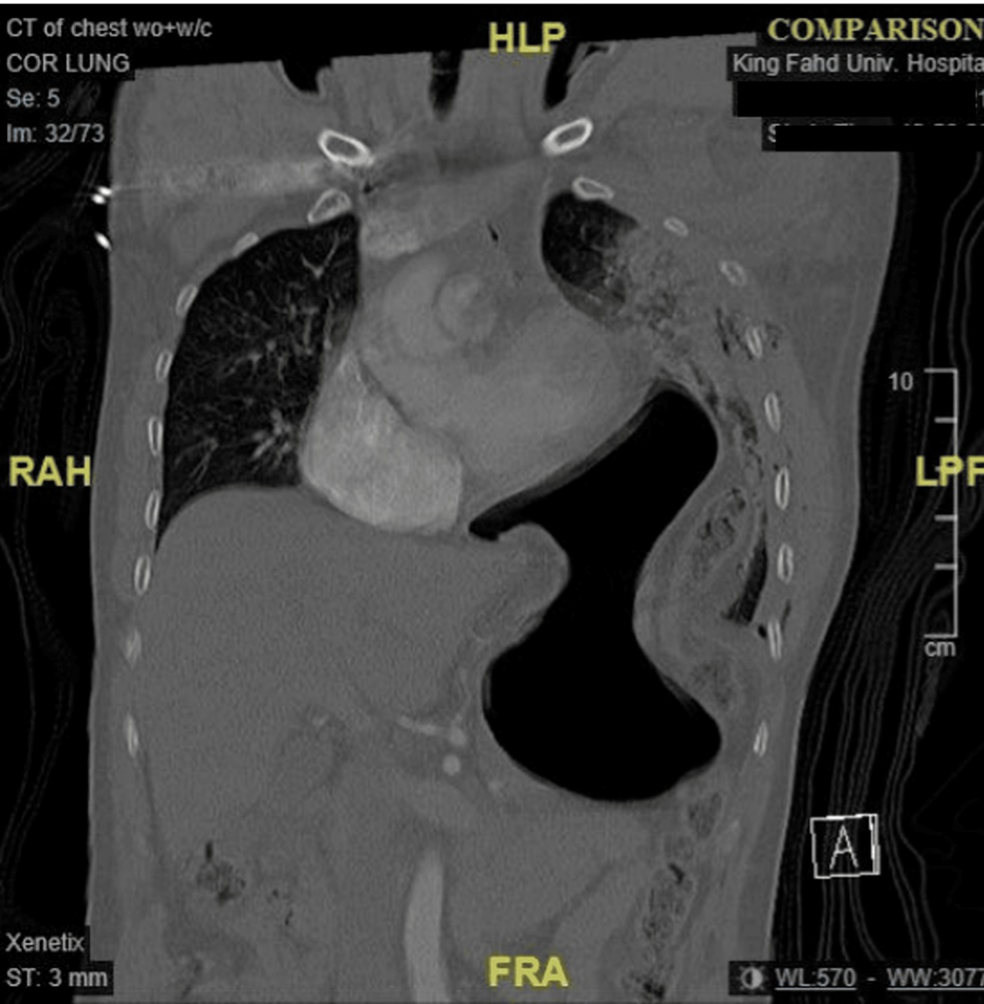
Left diaphragmatic rupture and herniation of the stomach and splenic flexure into the chest.

The patient’s blood pressure stabilized at 118/67 mmHg after a total of 3.5 liters of normal saline fluid was given, and he was then rushed to radiology to undergo a pan-body CT scan. The CT scan of the head without contrast came back with no evidence of intracranial trauma-related injuries; however, the CT scan of the abdomen and pelvis showed signs of left diaphragmatic rupture and herniation of the stomach and splenic flexure into the chest, in addition to multiple comminuted pelvic bone fractures (Figure 2).

**Figure 3:**
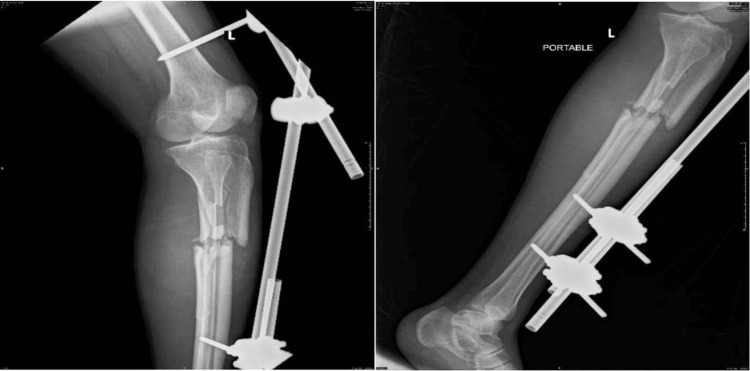
Left tibia and fibula open fracture treated by external fixation.

The patient was then taken to the operating room for an exploratory laparotomy, in which the left diaphragmatic injury and mesenteric tear were both repaired. The patient then underwent irrigation and debridement of the left tibia and fibula open fracture, and external fixation was applied due to extensive tissue damage (Figure 3).

During the operation, two units of packed red blood cells were given.

After surgical correction, the patient was taken to the surgical intensive care unit, where he remained sedated and intubated for the next 10 days, with a GCS of 3/15 throughout his stay. On the sixth day, the lung function of the patient began to deteriorate, and ventilator support was increased. On the tenth day, the patient passed away.

## Discussion

Traumatic diaphragmatic injury is a condition associated with both blunt and penetrating injuries to the thoracoabdominal region. It is believed to occur as a result of a sudden and forceful change in the pressure gradient between the thoracic and peritoneal cavities [12]. Approximately 3% of abdominal injuries are attributed to this type of injury, with a ratio of 2:1 for penetrating to blunt trauma. Left-sided injuries occur twice as frequently as right-sided injuries, likely due to the protective effect of the liver on the right hemidiaphragm [13]. While most cases are linked to high-velocity motor vehicle accidents, even accidents such as falls from heights, as in our case, can lead to this type of injury [1-3].

An important differential diagnosis that is more commonly found in the emergency setting is tension pneumothorax, which is characterized by the accumulation of air in the pleural cavity, leading to increased pressure. This condition operates with a unidirectional valve mechanism that hinders air from escaping the pleural cavity. It is associated with high mortality rates and significant hemodynamic instability upon presentation. The diagnosis of tension pneumothorax is primarily clinical, and any delay in diagnosis can have life-threatening consequences. This, in turn, may lead the physician to overlook an accompanying diaphragmatic injury.

Clinical features of diaphragmatic injury can vary depending on the timing of presentation after injury, the severity of other injuries, and the extent of herniation of abdominal organs into the thoracic cavity. This variability can lead to delayed diagnosis or inappropriate management of such cases. Symptoms may manifest immediately or even years after the initial injury [14]. In cases of acute trauma, patients presenting to the emergency department may experience abdominal pain, chest pain, and respiratory distress, which can mimic tension pneumothorax [9,12]. This misleading diagnosis may lead the treating physician to perform an unnecessary needle decompression based on clinical suspicion, which is usually performed as part of the primary survey preceding any imaging modality. In our patient, the constellation of decreased air entry on one side, no chest movement, and a history of trauma could have led to a misdiagnosis with the fatal consequence of a delay in treatment.

Diagnosing such injuries is challenging. A simple chest radiograph obtained during the primary survey is the quickest initial imaging step. However, it has been reported that chest radiographs were inconclusive in 59% of patients presenting with diaphragmatic injuries [12]. Radiographic signs may include irregularities in the diaphragm, mediastinal shift, and an elevated diaphragm, all of which may raise suspicion of diaphragmatic injury [8]. Additionally, the use of a nasogastric tube can aid in identifying defects in the diaphragm, enhancing the accuracy of the chest radiograph by visualizing bowel loops or the nasogastric tube in the chest [15].

Although not suitable for cases with severe, life-threatening hemodynamic instability, CT scans can provide valuable diagnostic information in stable conditions. It was found that CT scans led to a correct diagnosis in three out of nine patients [12]. The CT findings may include the "collar sign," discontinuity in the diaphragm, and intra-thoracic herniation of abdominal structures [12]. The diagnosis becomes even more challenging in patients receiving positive pressure ventilation, as this may prevent the bowel from herniating into the diaphragmatic defect and the thorax [1].

The presence of multiple thoracoabdominal injuries is highly suggestive of diaphragmatic injuries, where the best balance between sensitivity and specificity, according to one study, can be found in patients with intrusion ≥30cm combined with splenic, hepatic, or pelvic fractures and rib fractures with intrusion ≥30cm, generating the strongest sensitivity [16]. In our case, the presence of pelvic and rib fractures helped raise the suspicion of an underlying diaphragmatic rupture.

Delayed diagnosis and presentation are not uncommon, with the longest delayed presentation from the original time of trauma amounting to 50 years [17]. Possible complications of missed diaphragmatic hernias managed as tension pneumothorax include iatrogenic colonic perforation as a result of chest tube management. For cases of diaphragmatic hernias presenting similarly to tension pneumothorax, tension fecopneumothorax is a rare entity that occurs as a result of prolonged negative suction from a chest tube on an injured diaphragm, leading to subsequent herniation and perforation of the transverse colon [13].

In the past, this type of injury was only found before surgery in 43.5% of cases, discovered during surgery or autopsy in 41.3% of cases, and the remaining 14.6% were discovered late due to a lack of symptoms and clinical suspicion [12]. The appropriate measures to be taken in an emergency setting in hemodynamically unstable patients require rapid decompression of herniated gastrointestinal organs, achieved by placing the patient in an upright position and using a nasogastric tube, followed by surgical management in the operating room [2].

## Conclusions

In conclusion, our case of a middle-aged male patient who suffered a rare case of traumatic diaphragmatic hernia outlines the difficulty in diagnosing and ultimately managing it. A myriad number of differential diagnoses may arise from the constellation of symptoms presented in the mentioned case, with each next step playing a vital role in the outcome of the patient. This case, along with others reviewed in the literature, encourages physicians to include diaphragmatic injuries in the list of differential diagnosis of patients presenting with symptoms indicating tension pneumothorax. It also emphasizes the importance of using a chest radiograph in patients with enough hemodynamic stability to prevent subsequent iatrogenic colon perforation.
